# Exploring the Behavioral and Metabolic Phenotype Generated by Re-Introduction of the Ghrelin Receptor in the Ventral Tegmental Area

**DOI:** 10.3390/ijms18050914

**Published:** 2017-04-26

**Authors:** Louise J. Skov, Morten Jensen, Søren H. Christiansen, Cecilia Ratner, David P. D. Woldbye, Birgitte Holst

**Affiliations:** 1 Section for Metabolic Receptology, The Novo Nordisk Foundation Center for Basic Metabolic Research, University of Copenhagen, 2200 Copenhagen, Denmark; ljskov@sund.ku.dk (L.J.S.); mortenjensen@me.com (M.J.); cecilia.ratner@sund.ku.dk (C.R.); 2 Department of Biomedical Sciences, University of Copenhagen, 2200 Copenhagen, Denmark; 3 Center for Neuroscience, University of Copenhagen, 2200 Copenhagen, Denmark; schri@sund.ku.dk (S.H.C.); woldbye@sund.ku.dk (D.P.D.W.)

**Keywords:** ghrelin, ghrelin receptor, ventral tegmental area, energy expenditure, food anticipatory activity, stress, cocaine sensitivity

## Abstract

Ghrelin receptor (Ghr-R) signaling in neurons of the ventral tegmental area (VTA) can modulate dopaminergic function and the reward-related effects of both palatable foods and drugs of abuse. In this study, we re-introduced the Ghr-R in VTA neurons in Ghr-R knockout mice (Ghr-R^VTA^ mice) to specifically study the importance of the constitutively active Ghr-R for VTA neuronal signaling. Our results showed that re-introduction of the Ghr-R in the VTA had no impact on body weight or food intake under basal conditions. However, during novel environment stress Ghr-R^VTA^ mice showed increased food intake and energy expenditure compared to Ghr-R knockout mice, demonstrating the significance of Ghr-R signaling in the response to stress. Ghr-R^VTA^ mice also showed increased cocaine-induced locomotor activity compared to Ghr-R knockout mice, highlighting the importance of ghrelin signaling for the reward-related effects of activation of VTA neurons. Overall, our data suggest that re-introduction of the Ghr-R in the mesolimbic reward system of Ghr-R knockout mice increases the level of activation induced by both cocaine and novelty stress.

## 1. Introduction

The gut hormone ghrelin exerts many different functions, but one of its most well-described roles is the regulation of metabolism and food intake, largely mediated by the arcuate nucleus of the hypothalamus [1]. Reward-related appetite regulation, however, may be mediated through actions of ghrelin on the mesolimbic dopamine system that connects the ventral tegmental area (VTA) in the midbrain to the nucleus accumbens (NAc), a part of the ventral striatum [1,2,3,4].

The ghrelin receptor (Ghr-R) is highly expressed in neurons in the VTA [2,5] and injection of ghrelin into the VTA increases dopaminergic output to the NAc, as well as food intake [2,6]. Additionally, intra-VTA administration of a Ghr-R antagonist attenuates the ability of peripheral ghrelin to increase food intake, highlighting the importance of the mesolimbic system for the orexigenic effects of ghrelin [2]. Ghrelin also increases reward-seeking behavior and motivation towards obtaining palatable food [4,7,8] and drugs of abuse [9,10,11], presumably through action on dopaminergic neurons of the VTA. In humans, functional magnetic resonance imaging studies show that ghrelin increases the activity in regions involved in appetite regulation, including the mesolimbic dopamine system [12].

Independent of the orexigenic effect of ghrelin, Ghr-R signaling also affects the activity of the sympathetic nervous system to reduce energy expenditure and shift substrate utilization towards carbohydrates, thus stimulating the accumulation of fat [13,14,15]. The neurons responsible for these actions have not been finally determined, but intact β-adrenergic signaling is required [16] and hypothalamic paraventricular (PVN) neurons have been proposed as the link between the central nervous system and the sympathetic nervous system [17,18].

The Ghr-R has a high level of constitutive activity and signals with approximately 50% of its maximal capacity, depending on level of expression, in the absence of ghrelin, shown both in vitro and in vivo [19,20,21]. Thus, expression of the Ghr-R in the VTA leads to signaling that may be important independently of peripheral ghrelin secretion. We have previously demonstrated this effect, using a mouse model of virus-induced Ghr-R overexpression in the amygdala to evaluate the anxiolytic effect of Ghr-R signaling [22]. 

Knockout (KO) of the Ghr-R in mice results in normal or slightly reduced body weight and normal food intake under basal conditions [23,24]. On a high fat diet, Ghr-R ablation appears to be protective against diet-induced obesity [25,26]. This phenotype may be due to increased energy expenditure in combination with decreased food intake. Furthermore, Ghr-R KO mice display normal locomotor activity, but attenuated food anticipatory activity (FAA) after a feeding entrainment schedule [27,28,29]. Re-expression of the Ghr-R in dopaminergic neurons of Ghr-R KO mice partly restores ghrelin-induced food intake [30], underlining the significance of dopaminergic signaling for the orexigenic effect of ghrelin.

Cocaine very potently increases extracellular dopamine levels in the NAc, an effect that is augmented by ghrelin. Behaviorally, systemic and intra-NAc administration of ghrelin enhances cocaine-induced locomotor activity [31,32]. In contrast, treatment with a Ghr-R antagonist reduces cocaine-induced locomotor activity and NAc dopamine release [10]. Additionally, ghrelin KO mice are hypo-responsive to the effects of cocaine, reflected in attenuated locomotor activity following both acute and chronic cocaine injections, compared to wild-type mice [33]. These behavioral effects correlate with cocaine-induced changes in dopamine turnover in the striatum of wild-type mice that are not seen in ghrelin KO mice unless pre-treated with ghrelin [33].

Here, we examine the role of the Ghr-R in the VTA in the regulation of parameters demonstrated to be altered in Ghr-R KO mice, such as basic metabolism, food intake, and FAA, as well as parameters suggested to be VTA-dependent, e.g., cocaine-induced hyperactivity, and sucrose consumption. We re-introduce the Ghr-R in the VTA of mice that are completely deficient of the Ghr-R, using a recombinant adeno-associated virus (rAAV)-mediated approach. This method allows us to study novel properties of Ghr-R-mediated downstream signaling isolated in this particular region, as the receptor is not expressed in any other regions. Additionally, we study the expression of genes involved in regulation of the dopamine system in response to Ghr-R re-introduction. Importantly, the Ghr-R has a high constitutive activity and the receptor re-introduction on its own may be sufficient to exert an effect independent of ghrelin availability [19].

## 2. Results

### 2.1. Validation of Ghr-R Re-Introduction

**Figure 1:**
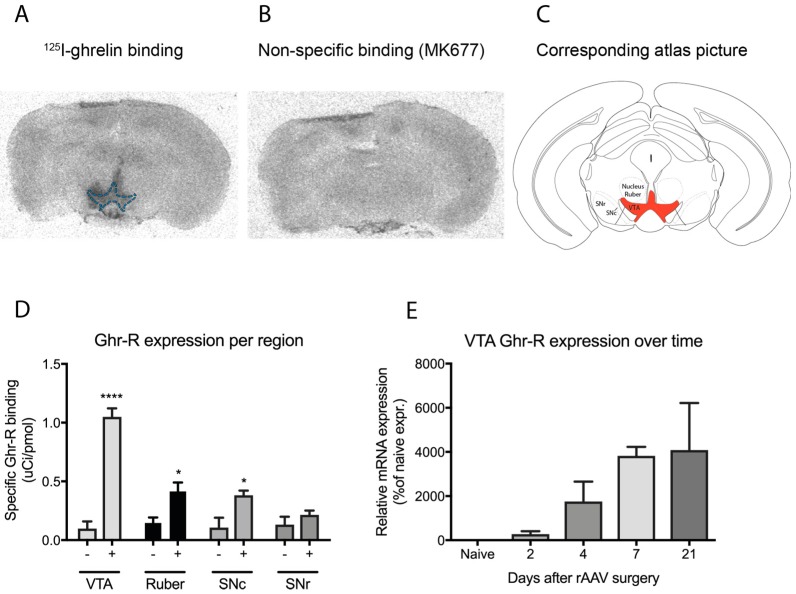
(**A**) Representative image of a brain section containing the ventral tegmental area (VTA; outlined in blue) from a ghrelin receptor (Ghr-R)^VTA^ mouse after autoradiography, showing specific binding of ^125^I-ghrelin to the Ghr-R; (**B**) Representative image of a brain section containing the VTA from a Ghr-R^VTA^ mouse after autoradiography, showing binding of non-labeled MK677, a Ghr-R agonist, to visualize non-specific binding of ^125^I-ghrelin; (**C**) The corresponding atlas picture with the VTA outlined in red, adapted from Paxinos & Keith 2001, figure 60 [36]; (**D**) Ghr-R expression of Ghr-R^VTA^ mice (+) and Ghr-R knockout (KO) control mice (−) in different regions of the midbrain, namely the VTA, nucleus ruber, substantia nigra pars compacta (SNc) and substantia nigra pars reticulata (SNr), *n* = 7; (**E**) Ghr-R expression in the VTA of Ghr-R^VTA^ mice at different time points after rAAV-mediated re-expression of the receptor in the VTA of Ghr-R KO mice, *n* = 2. * *p* ≤ 0.05, **** *p* ≤ 0.0001. All data are expressed as mean ± SEM.

To determine whether the rAAV-mediated re-introduction of the Ghr-R in the VTA of Ghr-R KO mice was successful, we performed autoradiography with ^125^I-ghrelin. The Ghr-R KO mice with rAAV-mediated re-introduction of the Ghr-R in the VTA are denoted as Ghr-R^VTA^ mice in this article, whereas Ghr-R KO mice with rAAV-mediated empty vector construct are denoted as Ghr-R KO mice or controls. The Ghr-R was able to bind the radiolabeled ^125^I-ghrelin in the VTA of the Ghr-R^VTA^ mice, demonstrating a successful re-introduction of the functional Ghr-R in the target area compared to Ghr-R KO control mice (Figure 1A–C). In addition, the receptor was also expressed at low levels in the nucleus ruber and substantia nigra pars compacta (SNc) located adjacent to the VTA (Figure 1D). We measured VTA Ghr-R expression at different time points after rAAV surgery and observed the expression to be increased from four days after virus administration (Figure 1E). rAAVs are efficient at providing long-term expression [34,35], so we estimated the Ghr-R expression to be stable throughout the remaining study period.

### 2.2. Food Intake, RER, and Energy Expenditure of Ghr-R^VTA^ Mice

**Figure 2:**
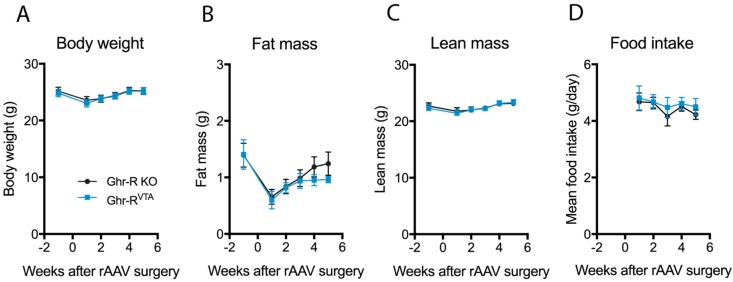
(**A**–**D**) Body weight, fat mass, lean mass, and daily food intake of Ghr-R^VTA^ mice and Ghr-R knockout (KO) control mice at different time points before and after recombinant adeno-associated virus (rAAV)-mediated re-introduction of the receptor in the VTA of Ghr-R KO mice. *n* = 7–8. All data are expressed as mean ± SEM.

During the first five weeks after the rAAV surgery, we monitored body weight, body composition, and food intake weekly, but observed no effect of Ghr-R re-introduction in the VTA on any of these parameters (Figure 2A–D). 

**Figure 3:**
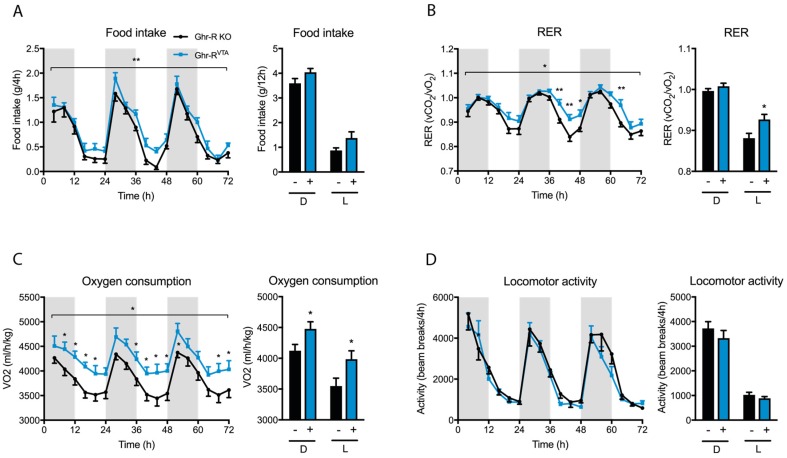
(**A**) Food intake per 4 h and food intake per dark (D) and light (L) cycle of Ghr-R^VTA^ mice (+) and Ghr-R knockout (KO) control mice (−) in the first three days in the indirect calorimetry cages during novel environment stress; (**B**–**D**) Respiratory exchange ratio (RER) values, oxygen consumption, and locomotor activity measurements during the same time period. * *p* ≤ 0.05, ** *p* ≤ 0.01. *n* = 7–8. All data are expressed as mean ± SEM. The stars depicted in the top of the graphs showing development over time indicate the main effects in a repeated-measures two-way ANOVA, whereas the stars depicted directly above the time points indicate significance for the given time point when tested post hoc by applying Fisher’s LSD test.

Following the initial five weeks after surgery we placed the animals in an indirect calorimetry system for metabolic measurements before initiating the FAA entrainment schedule. During the first three days in the novel environment of the calorimetric cages, we analyzed food intake, respiratory exchange ratio (RER), oxygen consumption (VO_2_) as a measure of energy expenditure, and locomotor activity of the animals. RER (VCO_2_/O_2_) is a measure of fuel utilization with a RER value of 1 indicating pure carbohydrate oxidation and an RER value of 0.7 indicating pure fat oxidation. We observed significantly-increased food intake, RER values, and VO_2_ in the Ghr-R^VTA^ mice, compared to Ghr-R KO control mice, with no difference in locomotor activity (Figure 3A–D). The increase in RER values could be explained by the increased food intake, as the food consisted of high-carbohydrate chow. The increased oxygen consumption, which is significantly increased during both dark and light phase, does not seem to be explained by an increase in activity level, as locomotor activity is similar in both Ghr-R^VTA^ mice and Ghr-R KO controls.

**Figure 4:**
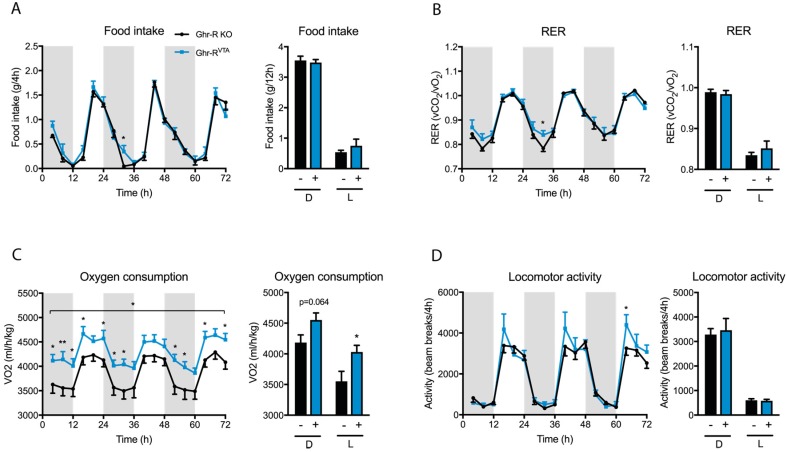
(**A**) Food intake per 4 h and food intake per light/dark cycle of Ghr-R^VTA^ mice (+) and Ghr-R knockout (KO) control mice (−) after 7–10 days in the indirect calorimetry cages after habituation as a measure of baseline values; (**B**–**D**) Respiratory exchange ratio (RER) values, oxygen consumption, and locomotor activity measurements during the same time period. * *p* ≤ 0.05, ** *p* ≤ 0.01, *n* = 7–8. All data are expressed as mean ± SEM. The stars depicted in the top of the graphs showing development over time indicate the main effects in a repeated-measures two-way ANOVA, whereas the stars depicted directly above the time points indicate the significance for the given time point when tested post hoc by applying Fisher’s LSD test.

After a total of seven days of habituation to the calorimetric cages we again analyzed food intake, RER, VO_2_, and locomotor activity for three days as a measure of stable baseline values. During these baseline measurements we observed no differences in food intake, RER, or locomotor activity between the two groups, while the Ghr-R^VTA^ mice still displayed a higher oxygen consumption compared to Ghr-R KO controls (Figure 4A–D).

Our results indicate that the Ghr-R^VTA^ mice had an altered response to a novel, stressful environment with a small increase in food intake, increased energy expenditure, and utilization of carbohydrate as an energy source. In a well-known environment after a habituation period, these values were normalized to levels comparable to the control mice. Only energy expenditure (VO_2_) remained elevated under basal conditions.

### 2.3. Food Anticipatory Activity of Ghr-R^VTA^ Mice

**Figure 5:**
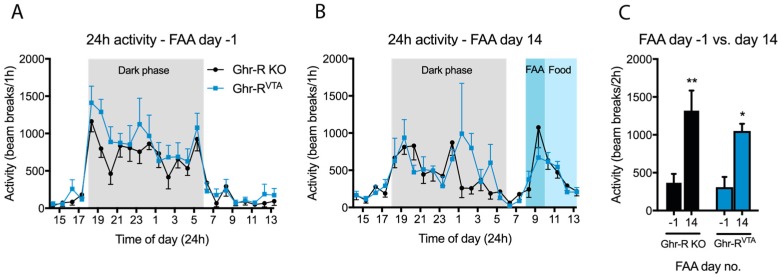
(**A**) 24 h activity of Ghr-R^VTA^ mice and Ghr-R knockout (KO) control mice measured as beam breaks per 1 h in indirect calorimetry cages before entrainment to a restricted feeding schedule; (**B**) 24 h activity of Ghr-R^VTA^ mice and Ghr-R KO control mice after 14 days of entrainment to restricted feeding schedule with food only available from 10:00–14:00 h (ZT 4–8). Food anticipatory activity (FAA) is measured as total beam breaks during the two hours before presentation of food (8:00–10:00 h; ZT 2–4); (**C**) The FAA of Ghr-R^VTA^ mice and Ghr-R KO control mice the day before the beginning of the entrainment schedule and after 14 days of restricted feeding measured as beam breaks per 12 h. * *p* ≤ 0.05, ** *p* ≤ 0.01, *n* = 7–8. All data are expressed as mean ± SEM.

After a total of 10 days of habituation to the calorimetric cages including novelty and baseline measurements we initiated 14 days of FAA entrainment, during which the mice only had access to food for 4 h each day from 10:00–14:00 h (ZT 4–8). Locomotor activity was monitored every day from 8:00–10:00 h (ZT 2–4) as a measure of the anticipatory activity leading up to the presentation of food [37,38]. We observed significant FAA after entrainment in both Ghr-R KO control mice and Ghr-R^VTA^ mice, but there was no difference in activity levels between the two groups (Figure 5A–C).

### 2.4. Cocaine-Induced Locomotor Activity of Ghr-R^VTA^ Mice

**Figure 6:**
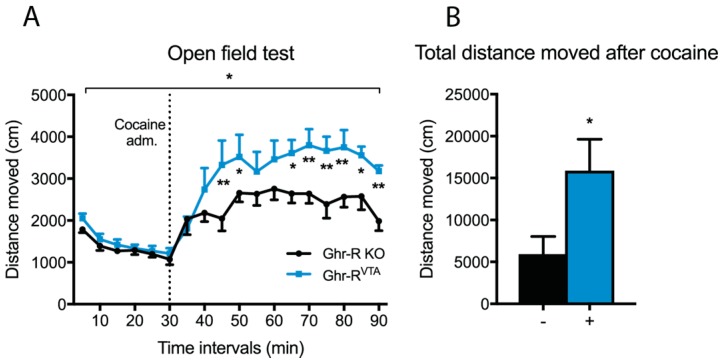
(**A**) Open field test of Ghr-R^VTA^ mice and Ghr-R knockout (KO) control mice as a measure of cocaine-induced hyperactivity. Locomotor activity is measured as the distance moved in 10-min intervals 30 min before (baseline) and 60 min after i.p. injection of cocaine. The star shown in the top indicates main effects in a repeated-measures two-way ANOVA, whereas the stars directly above the time points indicate the significance for the given time point when tested post hoc by applying Fisher’s LSD test. (**B**) The total distance moved by Ghr-R^VTA^ mice (+) and Ghr-R KO control mice (−) 0–60 min after i.p. injection of cocaine. * *p* ≤ 0.05, ** *p* ≤ 0.01, *n* = 7–8. All data are expressed as mean ± SEM.

We also examined whether Ghr-R re-introduction increased the sensitivity of the dopaminergic system in a more direct way by measuring cocaine-induced hyperactivity using the open field test. The mice were allowed 30 min of habituation to the open field before they were injected with cocaine (10 mg/kg, i.p.) followed by 60 min measurement of cocaine-induced hyperactivity. We observed a significantly increased cocaine-induced hyperactivity in the Ghr-R^VTA^ mice compared to Ghr-R KO control mice (Figure 6A,B).

### 2.5. Basal and Ghrelin-Induced Sucrose Consumption

**Figure 7:**
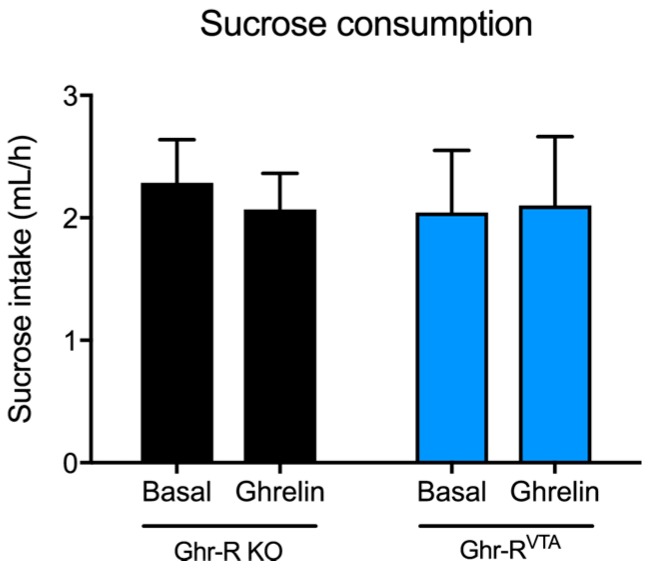
Sucrose consumption of Ghr-R KO mice and Ghr-R^VTA^ mice during basal conditions and 60 min after subcutaneous ghrelin administration. *n* = 7–8. All data are expressed as mean ± SEM.

To investigate the effect of re-introduction of the Ghr-R in the VTA on a food reward-related parameter, we measured sucrose consumption of Ghr-R^VTA^ mice and Ghr-R KO control mice under basal conditions and after a peripheral ghrelin injection. We observed no difference in sucrose consumption between the two groups neither under basal conditions nor one hour after ghrelin administration (Figure 7).

### 2.6. Expression of Dopaminergic Markers in the VTA of Ghr-R^VTA^ Mice

**Figure 8:**
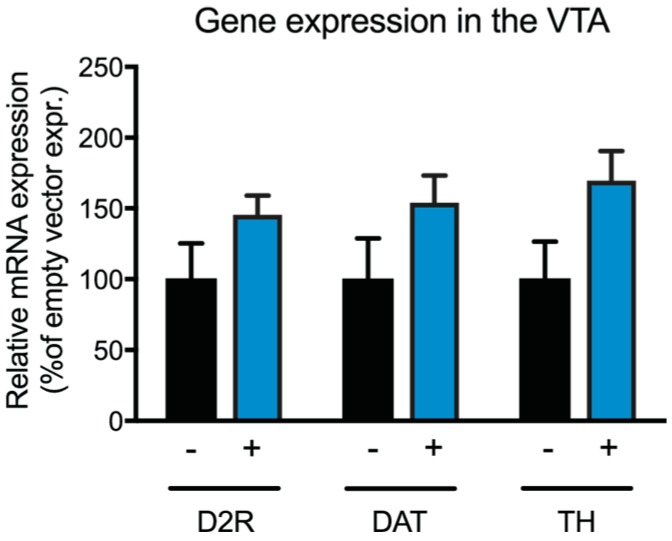
The relative gene expression in the VTA of Ghr-R^VTA^ mice (+) and Ghr-R knockout (KO) control mice (−) of dopaminergic markers measured by quantitative reverse transcription polymerase chain reaction (qRT-PCR) analysis of tissue samples obtained via laser capture microdissection. D2R: dopamine receptor D_2_; DAT: dopamine transporter; TH: tyrosine hydroxylase. *n* = 7–8. *p*-value TH = 0.067; *p*-value DAT = 0.159; *p*-value D2R = 0.159. All data are expressed as mean ± SEM.

We measured the expression of selected genes related to dopaminergic activity, synthesis, and reuptake after Ghr-R re-introduction in the VTA of Ghr-R KO mice. There was a trend towards increased expression of tyrosine hydroxylase (TH), the dopamine transporter (DAT), and the dopamine receptor D_2_ (D2R) in the Ghr-R^VTA^ mice compared to Ghr-R KO controls (Figure 8). However, the effect did not reach statistical significance for any of the genes.

## 3. Discussion

The focus of the present study was to examine the effects of re-introduction of the constitutively-active Ghr-R in the VTA of Ghr-R-deficient mice on parameters that are affected in Ghr-R KO mice and parameters regulated via VTA signaling. The purpose of this novel approach was to evaluate if expression of the Ghr-R in VTA neurons without any Ghr-R-mediated signaling in the rest of the body could modulate specific functions. Further, this approach allowed us to evaluate if the constitutive activity of the Ghr-R in the VTA had physiological in vivo functions. We investigated metabolism and food intake under stressed and basal conditions, FAA, cocaine-induced hyperlocomotion, and sucrose consumption. We found that, in response to a novel environment, the Ghr-R^VTA^ mice showed a trend towards increased food intake, and increased RER values, while their oxygen consumption was significantly higher, compared to Ghr-R KO control mice. Under basal conditions, after habituation to the new environment, food intake and RER values of Ghr-R^VTA^ mice normalized to levels similar to the control mice, whereas we also observed, under these conditions, increased oxygen consumption of Ghr-R^VTA^ mice compared to controls. We observed no difference in FAA or basal or ghrelin-induced sucrose consumption between Ghr-R^VTA^ mice and Ghr-R KO control mice, but Ghr-R^VTA^ mice had increased cocaine-induced hyper-locomotion and qPCR on VTA tissue samples showed a trend towards increased expression of dopaminergic markers.

We have previously, and successfully, used the model employed in this study, rAAV-mediated expression of the Ghr-R in a specific brain area, to study the anxiolytic effects of increased Ghr-R signaling in the amygdala of mice [22]. In our previous study, however, the Ghr-R overexpression was performed in wild-type mice whereas, in this study, we used Ghr-R KO mice. The advantage of using Ghr-R KO mice is that it allowed us to examine the effects of Ghr-R signaling, including constitutively-active ghrelin-independent signaling, in the midbrain, specifically, as the Ghr-R was only expressed in this region after rAAV surgery. Thus, we were able to dissect functions mediated by Ghr-R signaling specifically in the VTA, independent of endogenous Ghr-R signaling. We performed autoradiography with ^125^I-ghrelin to validate the expression of the Ghr-R and the Ghr-R agonist, MK677 to visualize specific binding of ^125^I-ghrelin by displacing it from Ghr-Rs [39]. This technique allowed us to verify that the receptor was functionally expressed at the protein level and able to bind ghrelin, thus providing a higher degree of validation than in situ hybridization, which is used in most studies.

We used a neuron-specific promoter for the Ghr-R expression. As the VTA contains both dopaminergic and γ-aminobutyric acid (GABA) neurons (approx. 30%) heterogeneously distributed within the VTA [40], it is very likely that some GABA neurons were targeted by the rAAV and, thus, expressed the Ghr-R. Abizaid et al. (2005) [2] estimate that approx. 60% of dopaminergic neurons and 30% of GABAergic neurons express the Ghr-R, and our approach may, thus, be more physiologically relevant than re-introducing the receptor in dopaminergic neurons only, as has previously been done [30]. However, we do not know the precise expression pattern of the Ghr-R in our study and how well it mimics the physiological expression pattern of the Ghr-R in the VTA. It is difficult to speculate on the importance of Ghr-R expression in GABA neurons in relation to our findings. As the main signaling pathway of the Ghr-R is through G_αq_ [41,42], increased expression of the receptor is likely to be associated with increased activation of the targeted neuron through increased cytosolic Ca^2+^ concentration. Increased activation of dopaminergic neurons will likely lead to increased reward signaling, whereas increased GABAergic signaling will lead to augmented inhibition of the neurons to which the GABA neurons project. These projections include local dopaminergic neurons of the VTA and cholinergic interneurons in the NAc [40] and increased activation of GABA neurons may reduce dopaminergic signaling. Activation of VTA dopaminergic neurons using a DREADD-G_q_ receptor increases locomotor activity in both mice and rats [43,44]. However, we do not observe increased locomotion in our study; hence, there appears to be a functional difference between activating dopaminergic neurons only and activating dopaminergic and GABAergic neurons in the VTA, perhaps due to increased inhibition from GABAergic neurons on dopaminergic neurons. Additionally, the short-term G_q_-selective neuronal activation induced by DREADD-G_q_ may mediate a different functional response, compared to the activation induced by the constitutively-active Ghr-R that signals through G_αq_, G_αi_, G_α12/13_, and β-arrestin recruitment [45].

As seen in Figure 1C, the virus-induced expression of the Ghr-R was found to leak into some areas surrounding the VTA, namely the nucleus ruber and SNc. Thus, it cannot be excluded that some of the effects we observed in this study were due to Ghr-R signaling in these areas. However, the expression of the receptor in these areas was much lower than in the VTA. Additionally, main functions of the nucleus ruber and SNc are motor control and locomotor activity, and we do not see any effect of the Ghr-R re-introduction on these functions. Thus, we estimate that the main contribution to the observed phenotype of the Ghr-R^VTA^ mice is Ghr-R re-introduction in neurons of the VTA.

During novel environment stress we observed increased food intake, RER values, and energy expenditure in Ghr-R^VTA^ mice compared to Ghr-R KO control mice, with no associated increase in locomotor activity. Introduction to a novel environment enforces stress upon mice and ghrelin is known to play a significant role in the stress response. Ghrelin secretion is potently stimulated by adrenergic agonists [46,47] mediated by binding to β1-adrenergic receptors expressed on the gastric ghrelin-producing cells [47]. Accordingly, ghrelin is released in response to both acute and chronic stress in rodents [48,49,50], possibly to counteract stress-induced anxiety and depression, as it has been demonstrated that ghrelin relieves anxiety and depressive behavior under stressed conditions [22,49]. Some studies show that stress induces hyperphagia and increased weight gain in rodents [30,51], whereas the opposite is the case in other studies [52,53]. Ghr-R KO mice show increased anxiety and depression when subjected to stress [49,54], highlighting the potential role of ghrelin as a defender against the negative effects of stress. In contrast, re-expression of the Ghr-R specifically in dopaminergic neurons of Ghr-R KO mice restores stress-induced hyperphagia and food-reward behavior, which may function as a protective coping mechanism against chronic stress [30]. The VTA has previously been suggested as a site for Ghr-R signaling to exert these putative protective effects in regard to stress [49]. Our results during novel environment stress indicate that re-introduction of the Ghr-R in the VTA may contribute to the minor stress-induced hyperphagia we observe in Ghr-R^VTA^ mice, but not in Ghr-R KO mice. As ghrelin levels increase during stress [48,49,50], it seems probable that Ghr-R^VTA^ mice have increased activation of VTA neurons during stress via Ghr-R signaling, leading to increased food intake. Hence, our results indicate that VTA Ghr-R signaling is involved in stress-mediated hyperphagia, although the difference in food intake between Ghr-R KO mice and Ghr-R^VTA^ mice is minor, though significant.

The increased RER values we observe in Ghr-R^VTA^ mice compared to controls in response to novelty stress indicate an increased preference for carbohydrate as energy source over fat, especially pronounced during the light phase. As the RER value increases along with increased food intake [55], the increased RER may be a reflection of the increased intake of high-carbohydrate chow observed in the Ghr-R^VTA^ mice. Normally, mice shift from mainly utilizing carbohydrate as an energy source during the active dark phase to primarily using fat as an energy source during the light phase when the mice sleep, reflected in decreased RER values during the light phase. 

The increased oxygen consumption without an associated increase in activity we observe in the Ghr-R^VTA^ mice during both novelty stress and under basal conditions may be due to an increased activation of the sympathetic nervous system with increased innervation of brown adipose tissue (BAT). Dopaminergic neurons in the PVN of the hypothalamus have been shown to increase BAT activation [56] but, to the best of our knowledge, no link between dopaminergic neurons of the VTA and energy expenditure has been described so far. However, evidence indicates that bidirectional connections exist between the VTA and the PVN [57,58], a region important for the regulation of energy expenditure. Neuronal projections to the VTA originate in a diverse collection of brain regions, with one of the most robust connections coming from the lateral hypothalamus (LH), where stimulation of projection neurons to the VTA induces feeding and reward-seeking behavior [59,60,61,62]. The VTA itself projects mainly to the NAc, prefrontal cortex, amygdala, and hippocampus [63]. The NAc has been suggested to project to the LH among many other regions [64], creating a complex and incompletely understood neurocircuitry. The effect on energy expenditure we observed when re-introducing the Ghr-R in the VTA of Ghr-R KO mice is unexpected and suggests a novel function for the VTA in the regulation of energy expenditure.

When food is only available during a restricted and predictable time of day, mice exhibit FAA, which involves an increase in locomotor activity preceding the presentation of food. Ghrelin levels are elevated prior to the presentation of food after entrainment to a restricted feeding schedule and ghrelin administration increases FAA [28]. It has previously been shown that Ghr-R KO mice display reduced FAA compared to wild-type mice [27,28,29] and acute and chronic treatment with a Ghr-R antagonist reduces FAA [29]. In our study, however, we observed similarly high levels of FAA in both the control Ghr-R KO mice and in the mice expressing the Ghr-R selectively in VTA neurons. These results indicate that Ghr-R signaling in the VTA is not sufficient to regulate the FAA response or that Ghr-R signaling is not important for FAA, as we observed substantial FAA in Ghr-R KO mice, contrary to other studies [27,28].

The Ghr-R^VTA^ mice showed increased cocaine-induced locomotor activity as compared to controls. It has previously been shown that seven days of systemic ghrelin does not change basal locomotor activity in rats, but it enhances the response to cocaine [65]. These results are in line with our findings that the Ghr-R^VTA^ mice show normal basal locomotor activity, but increased hyperlocomotion in response to cocaine administration. Our results suggest that chronic VTA Ghr-R expression is not enough to affect basal locomotor activity of Ghr-R KO mice. However, when the dopamine system was pharmacologically challenged with cocaine, we observed an increased sensitivity after VTA Ghr-R re-introduction. Thus, our results underline the importance of Ghr-R signaling in the VTA for the function of the dopamine system in relation to psychostimulants. In line with this, the acute hyperlocomotion- and conditioned place preference-inducing effects of ethanol, cocaine, amphetamine, and nicotine are attenuated or ablated by antagonism of the Ghr-R [9,10,66]. Heterodimerization between the Ghr-R and the dopamine receptor D_1_ (D1R) has been shown to amplify dopamine signaling in neurons that co-express the Ghr-R and the D1R [67] and this mechanism may contribute to the effect we observe. Supportive of increased dopaminergic signaling in Ghr-R^VTA^ mice, there was a trend towards increased expression of the dopaminergic markers, TH, DAT, and D2R in the VTA of these mice compared to controls, but the effects did not achieve statistical significance. Increased expression of enzymes involved in dopaminergic synthesis, reuptake, and activity is in line with the stimulatory effect ghrelin has on these neurons [68,69]. We did not observe any difference between Ghr-R^VTA^ mice and Ghr-R KO control mice on sucrose consumption under basal conditions, or after a peripheral administration of ghrelin. Thus, it appears that Ghr-R expression in the VTA is not sufficient to affect consumption of a rewarding substance. VTA ghrelin signaling has previously been implicated as a mediator of reward-related behavior in regards to palatable foods [4,7,8]. However, in previous studies, ghrelin has been administered directly into the VTA of wild-type mice with intact Ghr-R signaling throughout the brain. Our results suggest that the ability of peripheral ghrelin to induce increased intake of palatable substances requires intact Ghr-R signaling outside of the VTA.

In conclusion, we showed in this study that Ghr-R re-introduction in the VTA of Ghr-R KO mice increased food intake, RER, and oxygen consumption in response to novel environment stress. Only the effect on oxygen consumption was also present under basal unstressed conditions. Our findings indicate a new potential role for Ghr-R activation in the VTA to regulate energy expenditure directly or indirectly. Ghr-R re-introduction in the VTA of Ghr-R KO mice had no effect on food anticipatory activity, or sucrose consumption, but significantly increased cocaine-induced hyperlocomotion. These findings underline the importance of ghrelin signaling for the reward-related effects of activation of VTA neurons. 

## 4. Materials and Methods

### 4.1. Animals and Drugs

Male Ghr-R KO mice (C57BL/6J background, fully backcrossed, aged 8–11 weeks at the time of surgery, *n* = 7–8) [25] were used. All mice were socially housed in Macrolon cages until surgery, after which they were single housed for the duration of the study. Cages were enriched with housing and nesting material and kept at constant temperature (22 ± 2 °C) in a 12-h light/dark cycle with lights on at 6:00 h (ZT 0). Animals had free access to a chow diet (Altromin #1310, Brogaarden, Lynge, Denmark) and water unless otherwise specified. All experiments were approved by the Danish Animal Experimentation Inspectorate and conducted according to institutional guidelines (license no. 2012-15-2934-00054, approval 14 May 2009). Human ghrelin (Polypeptide Laboratories, Hillerød, Denmark) was dissolved in 0.9% saline and injected subcutaneously (s.c., 2 mg/kg). Cocaine (HS Pharmacy, Copenhagen, Denmark) was dissolved in 0.9% saline and injected intraperitoneally (i.p., 10 mg/kg). The drug solutions were prepared immediately before use.

### 4.2. Recombinant Adeno-Associated Virus Injections

The rAAV vectors (GeneDetect, Auckland, New Zealand) were derived from a mixture of serotypes 1 and 2. The vectors contained an inserted transgene, encoding the full-length cDNA for the human Ghr-R containing a FLAG polypeptide tag added to the N-terminus of the receptor (stock solution: rAAV-Ghr-R; 1 × 10^12^ genomic particles/mL). An empty vector without transgene was used as the control (rAAV-Ctrl; 1 × 10^12^ genomic particles/mL). The transgene was sub-cloned into an rAAV expression cassette, which consisted of the rat neuron-specific enolase promoter, the woodchuck post-transcriptional regulatory element and a bovine growth hormone polyA signal flanked by rAAV inverted terminal repeats. The injections were performed as previously described [70,71]. Briefly, the mice were pre-treated with a mixture of the analgesics Rimadyl (5 mg/kg, Pfizer, New York City, NY, USA) and Temgesic (0.06 mg/kg, Reckit Benckiser, Berkshire, UK) and the antibiotic Baytril (10 mg/kg, Bayer Healthcare, Leverkusen, Germany) before they were anaesthetized using isoflurane gas anesthesia (Baxter, San Juan, Puerto Rico). Following anesthesia, they were placed into a stereotaxic frame (Kopf Instruments, Tujunga, CA, USA), and a total volume of 1 μL viral vector suspension was injected through a glass pipette at 0.2 μL/min bilaterally into the VTA (anterior-posterior: −3.3 mm and medial-lateral: ±0.5 mm relative to Bregma; dorsal-ventral: 4.5 mm relative to skull surface, 1 μL at each injection site) [36]. After injection, the injection pipette was left in place for 3 min to minimize the backflow of viral particles through the injection track. Post-operatively, the mice received daily treatment with analgesic (Rimadyl, 5 mg/kg) and antibiotic (Baytril, 10 mg/kg) for two days.

### 4.3. Verification of rAAV-Mediated Re-Introduction of the Ghrelin Receptor

On the day of termination, the mice were decapitated and their brains removed and frozen on powdered dry ice. The brains were cut into 14 μm coronal sections through the VTA using a cryostat (Leica Microsystems CM3050 S, Wetzlar, Germany), mounted on Superfrost plus glass slides and kept at −80 °C until further use.

#### Autoradiography

The slides were thawed at room temperature and pre-incubated for 20 min in binding buffer (pH 7.4), containing 25 mM *N*-(2-hydroxyethyl)piperazine-N0-(2-ethanesulphonic acid) (HEPES), 2.5 mM CaCl_2_, 1 mM MgCl_2_, 0.5 g/L bacitracin, and 0.5 g/L bovine serum albumin. The slides were then incubated at room temperature for 60 min in binding buffer containing 0.15 nM [^125^I][His]-ghrelin (Perkin Elmer, NEX388, Waltham, MA, USA) to which 1 μM non-labeled MK677 (Tocris, Bristol, UK) was added to visualize specific binding. After a brief rinse, the slides were washed for 5 min in binding buffer at 4 °C and subsequently air-dried, before being exposed to ^125^I-sensitive Kodak Biomax MS films (Amersham Biosciences, Little Chalfont, UK) for four days at room temperature. The films were developed in a Kodak GBX developer.

### 4.4. Body Weight, Body Composition, and Food Intake

In order to observe a potential effect of the intra-VTA Ghr-R re-introduction on body weight and body composition, the mice were monitored weekly from just before rAAV surgery and throughout the first five weeks after surgery. Every week, the mice were weighed and their body composition analyzed by quantitative magnetic resonance imaging (MRI) using echo-MRI (Echo Medical Systems, Houston, TX, USA). At the same time the average weekly food intake was registered manually by weighing the content of the food hoppers.

### 4.5. Indirect Calorimetry

Following the five weeks of body composition measurements, oxygen consumption, food intake, RER, and locomotor activity of the mice were assessed using a 16-chamber indirect calorimetry system (PhenoMaster; TSE Systems, Bad Homburg, Germany) as previously described [55]. Briefly, measurements of food intake (g), RER (VCO_2_/VO_2_), oxygen consumption (VO_2_: mL/h/kg), and ambulatory activity (consecutive beam breaks) were sampled at the first three days of housing in the new calorimetric cages to evaluate novel environment stress and again after four additional days of acclimatization to evaluate baseline metabolism.

### 4.6. Food Anticipatory Activity

After 10 days of acclimatization and baseline measurement in the calorimetric cages the mice were put on scheduled feeding, with access to food only from 10:00 to 14:00 h (ZT 4–8) each day for 14 days with free access to water. Food intake and locomotor activity were measured continuously throughout the experiment. FAA was registered as total ambulatory activity (consecutive beam breaks) in the 2 h preceding food presentation (8:00–10:00 h; ZT 2–4) [37,38].

### 4.7. Open Field Locomotor Test

Four months after the FAA experiments, the mice were subjected to an open field locomotor test. The mice were placed in the center of the open field arena (40 cm × 40 cm with 65 cm walls) [71]. Baseline locomotor activity was measured for 30 min after which the mice were injected with cocaine (10 mg/kg, i.p.) and locomotor activity measured for 60 min. Each session was recorded and later scored for locomotor activity using Ethovision software (Noldus Information Technology, Wageningen, The Netherlands) by center-point detection.

### 4.8. Basal and Ghrelin-Induced Sucrose Consumption

Mice were moved to the experimental room 1 h before the start of the experiment and placed in an empty cage with only bedding material. Mice had access to water and a 10% sucrose solution. After 1 h of access, intake of water and sucrose solution was measured. On a separate day mice received an injection of ghrelin (2 mg/kg, s.c.) before being placed in the cage and intake was again measured after 1 h of access.

### 4.9. Expression of Dopaminergic Markers in the VTA

#### 4.9.1. Laser Capture Microdissection (LCM)

The VTA was collected bilaterally from three 14-μm coronal sections per animal on Superfrost plus glass slides. Brain sections were stained for 1 min with 0.1% cresyl violet acetate (Sigma-Aldrich, Brøndby, Denmark) dissolved in 70% EtOH. Sections were subsequently dehydrated briefly in 96% and 99.9% EtOH and finally dried at room temperature for at least 2 min. Using a PALM Microdissection instrument (Zeiss Microsystems, Jena, Germany), the VTA was identified and captured on Capsure Macro LCM Caps (Life Technologies, Applied Biosystems, Carlsbad, CA, USA).

#### 4.9.2. RNA Extraction and cDNA Synthesis from LCM Dissected Samples

Cell lysis and RNA extraction were performed using the RNeasy Micro Kit with DNase digestion according to the manufacturer’s instructions (Qiagen, Copenhagen, Denmark). The RNA was eluted in 14 μL RNase-free H_2_O and cDNA for qPCR analysis was produced using SuperScript III (Invitrogen, Nærum, Denmark).

#### 4.9.3. Quantitative Reverse Transcription (qRT)-PCR Analysis

We used a fixed cDNA volume of 0.83 μL cDNA per sample, diluted 1:6 in RNase-free H_2_O. RT-qPCR analysis was performed on a LightCycler480 (Roche Applied Science, Penzberg, Germany) using PrecisionPLUS Mastermix premixed with SYBRgreen (Primerdesign, Chandler's Ford, UK). The expression levels of the genes of interest were normalized to the average expression of the housekeeping genes TATA-box binding protein (TBP) and tyrosine 3-monooxygenase/tryptophan 5-monooxygenase activation protein, zeta (YWHAZ), using the ΔΔ*C*_t_ method [72]. All runs consisted of a pre-incubation step at 95 °C for 2 min followed by 45 cycles of 95 °C, 15 s; 60 °C, 45 s. The following forward (F) and reverse (R) primers were used: tyrosine hydroxylase (TH) F: CCT GGG AGA ACT GGG CAA ATG and R: CCG TCA TGC CTC CTC ACC TAT G; dopamine transporter (DAT) F: TGC TCT ACT TCA GCC TGT CG and R: TAT GCT CTG ATG CCA TCC AT; dopamine receptor D_2_ (D2R) F: GAC TCA ACA ACA CAG ACC AG and R: CGT AGA ACG AGA CGA TGG A. All analyses were performed in duplicate.

### 4.10. Statistics

All data was analyzed using GraphPad Prism 6.0 (Graphpad Software, San Diego, CA, USA). An unpaired two-tailed Student’s *t*-test was used when the means of two groups were compared. When more than two groups were compared, two-way analysis of variance (ANOVA) was used, followed by Fisher’s LSD post-hoc test for comparison of individual groups when appropriate. The level of significance was set at *p* ≤ 0.05 and all data are presented as mean ± standard error of the mean (SEM).
